# Novel POU3F4 variants identified in patients with inner ear malformations exhibit aberrant cellular distribution and lack of *SLC6A20* transcriptional upregulation

**DOI:** 10.3389/fnmol.2022.999833

**Published:** 2022-09-29

**Authors:** Emanuele Bernardinelli, Sebastian Roesch, Edi Simoni, Angela Marino, Gerd Rasp, Laura Astolfi, Antonio Sarikas, Silvia Dossena

**Affiliations:** ^1^Institute of Pharmacology and Toxicology, Paracelsus Medical University, Salzburg, Austria; ^2^Department of Otorhinolaryngology, Head and Neck Surgery, Paracelsus Medical University, Salzburg, Austria; ^3^Bioacoustic Research Laboratory, Department of Neuroscience, Biomedical Campus Pietro d’Abano, University of Padua, Padua, Italy; ^4^Department of Chemical, Biological, Pharmaceutical, and Environmental Sciences, University of Messina, Messina, Italy; ^5^Interdepartmental Research Center of International Auditory Processing Project in Venice (I-APPROVE), Department of Neurosciences, University of Padova, Santi Giovanni e Paolo Hospital, ULSS3, Venice, Italy

**Keywords:** POU3F4, DFNX2, DFN3, incomplete partition 3, hearing loss, enlarged vestibular aqueduct, *SLC6A20*

## Abstract

Hearing loss (HL) is the most common sensory defect and affects 450 million people worldwide in a disabling form. Pathogenic sequence alterations in the *POU3F4* gene, which encodes a transcription factor, are causative of the most common type of X-linked deafness (X-linked deafness type 3, DFN3, DFNX2). POU3F4-related deafness is characterized by a typical inner ear malformation, namely an incomplete partition of the cochlea type 3 (IP3), with or without an enlargement of the vestibular aqueduct (EVA). The pathomechanism underlying POU3F4-related deafness and the corresponding transcriptional targets are largely uncharacterized. Two male patients belonging to a Caucasian cohort with HL and EVA who presented with an IP3 were submitted to genetic analysis. Two novel sequence variants in *POU3F4* were identified by Sanger sequencing. In cell-based assays, the corresponding protein variants (p.S74Afs*8 and p.C327*) showed an aberrant expression and subcellular distribution and lack of transcriptional activity. These two protein variants failed to upregulate the transcript levels of the amino acid transporter gene *SLC6A20*, which was identified as a novel transcriptional target of POU3F4 by RNA sequencing and RT-qPCR. Accordingly, *POU3F4* silencing by siRNA resulted in downregulation of *SLC6A20* in mouse embryonic fibroblasts. Moreover, we showed for the first time that SLC6A20 is expressed in the mouse cochlea, and co-localized with POU3F4 in the spiral ligament. The findings presented here point to a novel role of amino acid transporters in the inner ear and pave the way for mechanistic studies of POU3F4-related HL.

## Introduction

Congenital hearing loss (HL) is estimated to affect 1 in 1,000 newborns, is the most common sensory defect and affects 450 million people worldwide in a disabling form (Sheffield and Smith, 2019). In developed countries, 60–80% of the reported cases of HL are estimated to result from genetic defects (Shearer et al., 2017), which mostly lie in autosomal genes and in about 1–5% of the cases in genes on the X chromosome (Del Castillo et al., 2022). Among the four types of X-linked HL (DFN2, DFN3, DFN4, and DFN6), X-linked deafness type 2 (DFNX2, also known as DFN3, OMIM #304400) accounts for about 50% of X-linked deafness cases and is associated with pathogenic sequence alterations in the *POU3F4* gene (*BRAIN-4*, *BRN4*, OMIM *300039) (Phippard et al., 1999), which codes for a highly conserved protein belonging to the POU family of transcription factors.

Hereditary HL is often associated with inner ear malformations, which are detectable in about 20% of the patients affected by sensorineural HL (SNHL) (Sennaroglu and Bajin, 2017). The observed abnormalities range from an enlargement of the vestibular aqueduct (EVA) to an incomplete partition of the cochlea (IP) or a complete labyrinthine aplasia, where cochlea and vestibule are completely absent. The most commonly detected abnormality is an EVA, with or without cochlear malformations. EVA can be associated with defects in multiple genes, the most frequently involved being *SLC26A4*, which codes for the anion exchanger pendrin (OMIM *605646). Other genes that have been found mutated in patients with EVA code for the pendrin transcription factor FOXI1 (OMIM *601093), the potassium channel KCNJ10 (OMIM *602208), the gap junction protein connexin-26 (OMIM *121011), and the transcription factor POU3F4 (Roesch et al., 2021).

Pathogenic sequence alterations in *POU3F4* result in a mixed type of HL (conductive and sensorineural), often progressive, associated with characteristic bone abnormalities (de Kok et al., 1995). In this context, a computed tomography (CT) scan of the temporal bone typically reveals a dilation of the internal acoustic canal (IAC) and a malformation of the cochlea, defined as an incomplete partition of type 3 (IP3), with or without an EVA. IP3 is characterized by the absence of the bony modiolus and the septum at the base of the cochlea, while the *interscalar septum* is still partially present. Also, often observed in DFN3 patients are an abnormal calcification and fixation of the stapes and a gusher of cerebrospinal fluid upon opening of the labyrinth during cochlear implant surgery (Sennaroglu and Bajin, 2018).

The transcription factor POU3F4 belongs to the POU superfamily of transcription factors, which are characterized by two DNA binding domains, the POU-specific domain and the POU-homeodomain, both determining the specificity of the binding to target genes (Lee et al., 2009; Malik et al., 2018). Despite a limited knowledge regarding the actual transcriptional targets of POU3F4, its role in the development of the hearing apparatus is well established. During embryonic development of the inner ear, POU3F4 is mainly expressed in the otic mesenchyme surrounding the otic vesicle (Phippard et al., 1999) and plays a role in the development of the otic neural tube and the bony tissue surrounding the auditory-vestibular system, possibly by regulating the expression of ephrin type-A receptor 4 (*EphA4*) and Ephrin-B2 (*Efnb2*) (Coate et al., 2012; Raft et al., 2014). Alterations in the spiral ganglion axon fasciculation and synapse formation observed in *Pou3f4^y/–^* hemizygous male mice were associated to a lack of EphA4 expression, while the ossification defects of the stapes and other temporal bone structures were linked to a defective regulation of the Ephrin-B2 gene.

In neonatal wild type mice, the highest expression of *Pou3f4* in the inner ear was found in the spiral ligament and remarkable pathological changes were observed in the spiral ligament fibrocytes in *Pou3f4-*deficient mice (Minowa et al., 1999). Spiral ligament fibrocyte adhesion is essential for cochlear K^+^ recycling and maintenance of the endocochlear potential and hence the hearing function. Lack of POU3F4 expression in the development of the mouse inner ear leads to a defective adhesion of the fibrocytes in the spiral ligament and consequent degeneration of Cx26/Cx30 gap junction plaques in the inner sulcus (Kidokoro et al., 2014), thus explaining the loss of the endocochlear potential and deafness in *Pou3f4-*deficient mice. Outside of the hearing organ, POU3F4 is mainly expressed in brain (Wu et al., 2021), kidney and pancreas (Naranjo et al., 2010; Tantin, 2013), but its functional role in these organs has not been completely established.

The present study was guided by the genetic appraisal of two male patients with mixed HL and IP3 belonging to our Austrian cohort of patients diagnosed with EVA (Roesch et al., 2018). Two novel *POU3F4* sequence variants leading to aberrant protein products were identified in these patients. The two pathogenic POU3F4 protein variants failed to upregulate *SLC6A20*, which was identified as a novel transcriptional target of POU3F4.

## Materials and methods

### Patient recruitment

Two male subjects diagnosed with HL associated with inner ear malformations (EVA and IP3, Table 1) were enrolled in the study. The research was prospectively reviewed and approved by a duly constituted ethics committee (415-E/2092/6-2017, 9 May 2017) and has therefore been performed in accordance with the principles embodied in the 1964 Declaration of Helsinki and its later amendments.^1^ Written informed consent was obtained from the subjects or their legal representatives prior to blood sampling and genetic testing. For both patients, imaging studies of the inner ear by computed tomography (CT) of the temporal bones were performed. EVA was defined as an enlargement of the vestibular aqueduct >1.5 mm midway between the endolymphatic sac and the vestibule and fitted the definition criteria for EVA (Valvassori and Clemis, 1978; Vijayasekaran et al., 2007) in both patients. An IP3 was identified as a cochlea of normal size lacking the bony modiolus and the septum at the base of the cochlea, while the *interscalar septum* was partially present (Sennaroglu and Bajin, 2018). Characterization of HL was based on side-specific pure-tone audiometric testing, as well as the patient’s history. Related to the time of speech development, the onset of HL was defined as congenital, prelingual, perilingual or postlingual. Clinical course of HL with possible hearing drops was obtained through audiometric tests and patient’s history. Surgical reports were screened for intraoperative gusher phenomenon, defined as a visible efflux of cerebro-spinal fluid (CSF) after cochleostomy, during cochlear implant surgery.

**TABLE 1:** Summary of the clinical finding of the two index patients.

Patient ID	Ethnicity	Sex	Age (year of birth)	EVA, side	IP3, side	Side affected by HL	Degree of HL	CSF gusher during CI surgery
#569	Caucasian	M	33 (1989)	Bilateral	Bilateral	Both	Severe	Yes, both sides
#667	Caucasian	M	19 (2003)	Left	Bilateral	Both	Right, profound Left, severe	Yes, right side

CI, cochlear implant; CSF, cerebrospinal fluid; EVA, enlarged vestibular aqueduct; HL, hearing loss; IP3, incomplete partition of the cochlea type 3.

### Genomic DNA analysis

Patient whole blood was collected via venipuncture in plastic tubes with potassium-ethylenediaminetetraacetic (EDTA) acid as the anticoagulant. Total genomic DNA (gDNA) was purified from ∼350 μl blood with the EZ1^®^ DSP DNA Blood kit (QIAGEN, Hilden, Germany) using the EZ1 Advanced XL platform (QIAGEN) according to the manufacturer’s instructions. Quantification was performed with the QIAxpert (QIAGEN) spectrophotometer. Only samples with an absorbance 260/280 between 1.7 and 1.9 were used for downstream analysis. The coding exons and no less than 50 nucleotides of the intro-exon boundaries of *POU3F4*, *GJB2*, *FOXI1* and *KCNJ10* were amplified by end-point polymerase chain reaction (PCR) with the primers indicated in Supplementary Table 1. For *POU3F4*, 50 μl PCR reaction contained 1X JumpStart REDAccuTaq Long and Accurate (JS RAT LA) DNA Polymerase buffer (Sigma-Aldrich, St. Louis, MO, USA), 0.3 mM dNTPs (Thermo Fisher Scientific, Waltham, MA, USA), 5% dimethyl sulfoxide (Sigma-Aldrich), 0.4 μM forward and reverse primers, 1.5 units JS RAT LA DNA Polymerase (Sigma-Aldrich) and 100 ng gDNA template. For the other genes, the PCR reaction was similar, with minor modifications. For *SLC26A4*, primers and procedure were formerly described (Roesch et al., 2018). Five μl of each PCR product was run on a 1% agarose gel for size verification, the remnant was purified with the QIAquick PCR purification kit (QIAGEN) and Sanger sequenced in the forward and reverse orientations (Microsynth AG, Switzerland) with the primers indicated in Supplementary Table 1. The resulting sequences were compared against the NCBI *Homo sapiens POU3F4* (OMIM ID: *300039, GenBank ID: NG_009936.2), *GJB2* (OMIM ID: *121011, GenBank ID: NG_008358.1), *SLC26A4* (OMIM ID: *605646, GenBank ID: NG_008489.1), *FOXI1* (OMIM ID: *601093, GenBank ID: NG_012068.2), and *KCNJ10* (OMIM ID: *602208, GenBank ID: NG_016411.1) genomic reference sequence assembly. The presence of the two large genomic deletions del(GJB6-D13S1830) and del(GJB6-D13S1854) was verified by multiplex PCR according to the method described in detail by del Castillo (del Castillo et al., 2005).

### Plasmid constructs

A pFLAG-CMV™-4 vector (Sigma-Aldrich), containing the coding sequence (CDS) of wild type or mutated human *POU3F4* cloned from human genomic DNA, was used for subcellular localization analysis by immunocytochemistry and confocal microscopy. Following transfection of cells with this construct, proteins are produced with a N-terminal FLAG epitope (DYKDDDDK). Alternatively, the pEYFP-C1 vector (Clontech, Mountain View, CA, USA) was used for these experiments. In this case, POU3F4 is produced with the enhanced yellow fluorescent protein (EYFP) fused to its N-terminus.

For dual luciferase assay experiments, the *POU3F4* CDS was sub-cloned into a pIRES2-EGFP vector (Clontech). With this construct, an untagged protein product was co-expressed with the transfection marker enhanced green fluorescent protein (EGFP) as two individual proteins. pGL4.11 POU3F4prom–475 + 25 reporter construct for the assay was produced by sub-cloning a fragment of *POU3F4* own promoter (Lee et al., 2009) upstream of the coding sequence of firefly luciferase in a pGL4.11 vector. The pRL-CMV vector, coding for renilla luciferase, was used for the normalization of firefly luciferase signal.

Sequence alterations in the CDS of *POU3F4* were obtained using the QuikChange^®^ Site-directed mutagenesis kit (Stratagene, San Diego, CA, USA). The sequence of all plasmid inserts was verified by Sanger sequencing (Microsynth AG, Balgach, Switzerland).

### Cell culture and transfection

HEK293Phoenix (DiCiommo et al., 2004) and HeLa (CCL-2, American Type Culture Collection, ATCC, Manassas, VA, USA) cells were cultured in Dulbecco Modified Eagle’s Medium (DMEM) supplemented with 2 mM L-glutamine, 1 mM sodium pyruvate, streptomycin-penicillin and 10% fetal bovine serum (FBS). Mouse embryonic fibroblasts (MEFs, CRL-2991, ATCC) were cultured in high glucose DMEM supplemented with 2 mM L-glutamine, 1 mM sodium pyruvate, streptomycin-penicillin and 10% FBS. Cell cultures were kept in a humidified incubator at 37°C and 5% CO_2_.

Transient transfection of HEK293Phoenix and HeLa cells was performed either with the calcium-phosphate co-precipitation method or with Metafectene PRO (Biontex, Munich, Germany) respectively, as previously described (De Moraes et al., 2016).

For *POU3F4* silencing experiments, MEFs were transfected with Metafectene SI + (Biontex) according to the manufacturer’s instructions.

### Dual luciferase assay

For the dual luciferase assay, HEK293Phoenix cells were seeded in twenty-four-well plates and transiently transfected on the following day with 250, 250, and 20 ng of wild type or mutated pIRES2-EGFP POU3F4 or empty pIRES2-EGFP, pGL4.11 POU3F4prom-472 + 25, and pRL-CMV vectors, respectively. Twenty-four hours later, media containing the transfection mix was replaced with fresh media. Forty-eight hours after transfection, the cells were prepared for the assay using the Dual Luciferase^®^ Assay System (Promega, Madison, Wisconsin, USA). Media was removed and cells were washed once with phosphate buffered saline (PBS, 136.89 mM NaCl, 2.69 mM KCl, 3.21 mM Na_2_HPO_4_, pH 7.4). Passive lysis buffer (200 μl) was added to each well and cells were incubated at room temperature (RT) for 15 min with shaking, followed by 15 min at -20°C and 15 min at room temperature with shaking. Twenty μl lysate were transferred in the wells of a white ninety-six-well plate for the assay. Fifty μl of luciferase assay reagent (LAR) II were added to each well and luminescence from the Firefly luciferase was detected for 10 s. Fifty μl of STOP & Glo reagent were then added to simultaneously quench the Firefly luciferase signal and activate the Renilla luciferase signal. The Renilla luciferase luminescence was also detected for 10 seconds. Addition of the LARII and STOP & Glo buffers, as well as detection of the luminescence was performed using the VICTOR™ X3 2030 multi-label reader (Perkin Elmer, Waltham, MA, USA).

### Subcellular localization analysis with fluorescent fusion proteins

HeLa cells were seeded into 6-well plates, grown overnight, transfected with 1.5 μg of plasmid DNA, transferred on glass slides for microscopy twenty-four hours post-transfection, fixed in 4% paraformaldehyde and imaged forty-eight hours post-transfection. Subcellular localization of POU3F4 variants was determined by co-localization between wild type or mutant POU3F4 with EYFP fused to the N-terminus and 4’,6-Diamidino-2-Phenylindole (DAPI), as a marker of the nuclear compartment. Co-localization was detected and quantified as previously described (De Moraes et al., 2016). Shortly, imaging was performed by sequential acquisition with a Leica TCS SP5II AOBS confocal microscope (Leica Microsystems, Wetzlar, Germany) equipped with an HCX PL APO 63x/1.20 Lambda blue water immersion objective and controlled by the LAS AF SP5 software (Leica Microsystems). EYFP was excited with the 514 nm line of the Argon laser and emission was detected in the 525–600 nm range; DAPI was excited at 405 nm with a diode laser and emission was detected in the 420–485 nm range. Co-localization was quantified and expressed as the Pearson’s correlation coefficient (Adler and Parmryd, 2010), overlap coefficient and co-localization rate.

### RNA sequencing

HEK293Phoenix cells were transiently transfected with pIRES2-EGFP POU3F4 wild type, POU3F4 p.C327* or the empty pIRES2-EGFP vector as a control. Forty-eight hours after transfection, total RNA was extracted with the RNeasy Micro Kit (QIAGEN) according to the manufacturer’s instruction and quantified. Samples were submitted for RNAseq analysis to the Bioinformatics Core Facility, Centre for Molecular Medicine, CEITEC Masaryk University, Brno, Czech Republic. Raw reads were quality checked, pre-processed and mapped to the reference genome with gene annotation (genome version: Ensembl GRCh38) with multiple tools (FastQC, MultiQC STAR, Samtools). Mapped reads were counted and summarized to genes, whereby only uniquely mapped and uniquely assigned reads were counted. The following checks were performed to estimate the overall sample quality: rRNA content estimate (fastqflscreen), read duplication rate (dupRadar, Picard tools), sequenced (targeted) regions (RSeQC, Picard tools), 5’/3’ coverage bias (Picard tools), expressed gene biotypes (featureCounts) and library strandedness (RSeQC). Differential genes expression was calculated (edgeR, DESeq2) by comparing all conditions against each other. Gene expression analysis was then manually refined by selecting the significantly upregulated genes.

### Western blot

HEK293Phoenix cells for Western blot were transfected with pIRES2-EGFP POU3F4 wild type, POU3F4 p.C327* or the empty pIRES2-EGFP vector. Total protein extraction was performed by denaturing lysis of cells twenty-four hours after transfection. Protein extracts (20 μg) were electrophoresed with constant voltage (120 V) on 12% SDS-PAGE gels. Proteins were transferred overnight onto polyvinylidene fluoride (PVDF) membranes with constant amperage (0.25 mA). The membranes were blocked for 1 h at room temperature in 5% non-fat dry milk in Tris-buffered saline containing 0.01% Tween 20 (TBST). Membranes were incubated overnight at 4°C with the primary antibody (rabbit anti-POU3F4, 711871, Thermo Scientific) diluted in 5% non-fat dry milk in TBST, washed 3 times in TBST and incubated for 1 h at room temperature with the secondary antibody in TBST containing 5% non-fat dry milk. Immunocomplexes were visualized using the ODYSSEY infrared imaging system (LICOR, Lincoln, NE, USA). The membrane was then stripped with a buffer containing 25 mM Glycine, 2% SDS, pH 2 and blotted again with anti-glyceraldehyde-3-phosphate dehydrogenase (GAPDH) antibodies (goat anti-GAPDH, A00191, GenScript, Piscataway, NJ, USA) following the same procedure described above. The anti-rabbit (926-32211, 1:20000 dilution) and the anti-goat (926-32214, 1:20000 dilution) IRD-800-CW secondary antibodies were from LICOR.

### RNA extraction and real time PCR analysis

Total RNA from transfected cells was extracted with RNeasy Micro Kit (QIAGEN) according to the manufacturer’s instructions and quantified with the QIAxpert (QIAGEN) spectrophotometer.

Total RNA from mouse kidney, small intestine, and lung were from commercial sources (Clontech and AMS Biotechnology Ltd., Abingdon, United Kingdom).

Total RNA extraction from mouse cochlea was performed as following: cochleae were explanted from 2-month-old euthanized wild type C57BL/6J mice and preserved in RNA-Protect solution (QIAGEN) until total RNA extraction was performed. For RNA extraction, RNA-Protect was removed and lysis of the whole cochlea was performed with the Tissue Lyser instrument (QIAGEN) in a QIAzol solution (QIAGEN). Debris were removed by centrifugation and phases were separated by centrifugation after the addition of chloroform. The upper phase was then transferred to a RNeasy MinElute spin column for further purification steps following the protocol provided by the manufacturer (QIAGEN). RNA was quantified with the QIAxpert (QIAGEN) spectrophotometer.

Retrotranscription of the RNA extracted either from cells or from tissues was performed with the QuantiTect Reverse Transcription Kit (QIAGEN) with 1 μg RNA per reaction, according to the manufacturer’s instructions. Real Time PCR (RT-qPCR) was performed on the Rotor-Gene Q instrument (QIAGEN) using the GoTaq™ qPCR Master Mix (Promega). Primers were designed for the specific amplification of *POU3F4* or *SLC6A20* transcripts (Supplementary Table 2). The indicated housekeeping genes were used for normalization of mRNA abundance. Relative mRNA levels were calculated using the comparative threshold cycle method (ΔΔCT).

### Immunohistochemistry and immunocytochemistry

Cochlea sections for immunohistochemistry were prepared by fixing mice (CBA/J, 12-week-old) whole cochleae in Shandon Glyo-Fixx (Thermo Scientific) overnight at 4°C. Temporal bones were decalcified with Surgipath Decalcifier I (Leica Biosystems) for 20 h at 4°C followed by Surgipath Decalcifier II for 3 h at room temperature. Decalcified cochleae were included in paraffin with an automatic processor and manual embedding system (SLEE medical GmbH, Nieder-Olm, Germany). Paraffin embedded samples were sectioned with a thickness of 5 μm with a semiautomatic microtome (Thermo Scientific). After paraffin removal and rehydration of the sections, antigen retrieval was performed in sodium citrate buffer (pH 6) for 10 min at 95°C. Endogenous peroxidases were inactivated with a 1% hydrogen peroxide solution and tissue sections were blocked in normal goat serum for 45 min at room temperature before incubation with primary antibodies (rabbit polyclonal anti-POU3F4, P100907_T100, Aviva System Biology, diluted 1:200, or rabbit polyclonal anti-SLC6A20, PA5-104153, Invitrogen, diluted 1:200). Incubation with primary antibodies was performed at 4°C for 16 h. Immunocomplexes were detected with the ABC-kit system (Vector Laboratories) and DAB chromogen (Dako, Agilent, Santa Clara, CA, USA). Nuclei were stained with hematoxylin staining (Leica Biosystems). Slices were finally dehydrated and mounted with Surgipath Micromount (Leica Biosystems) before imaging with an optical microscope ECLIPSE 50i (Nikon Europe, Amstelveen, Netherlands). Images were acquired with the Nis Elements D 3.2 software (Nikon).

Immunocytochemistry was performed as previously described (Meyer et al., 2004; Dossena et al., 2006) with minor modifications. Shortly, HeLa cells were transfected with N-terminally flagged wild type POU3F4, POU3F4 p.S74Afs*8 or POU3F4 p.C327* for 48 h, fixed in Hank’s Balanced Salt Solution (HBSS) containing 4% paraformaldehyde for 30 min, permeabilized with HBSS containing 0.2% Triton X-100 for 15 min, blocked with 3% bovine serum albumin (BSA) in HBSS for 1 h at room temperature and incubated overnight at 4°C with a mouse monoclonal anti-FLAG antibody (Sigma-Aldrich) diluted to 20 μg/ml in HBSS containing 0.1% BSA. Next, cells were incubated for 1 h at room temperature with an Alexa Fluor^®^ 488-conjugated goat anti-mouse antibody (Invitrogen Life Technologies, Carlsbad, CA, USA), counterstained with DAPI, mounted in MOWIOL^®^ 4-88 (Sigma-Aldrich) and imaged as described above for the co-localization of the fluorescent signal with the cell nucleus by confocal microscopy.

### Statistical analysis

All data are expressed as arithmetic means ± SEM and analyzed with GraphPad Prism software (version 5.00 for MacOSX, GraphPad Software, San Diego, CA, USA). Significant differences between data sets were verified by one-way Analysis of Variance (ANOVA) with Bonferroni’s post-test or by unpaired Student’s t-test, as appropriate. A *p* value <0.05 was considered as statistically significant; (*n*) corresponds to the number of independent measurements.

## Results

### Clinical features of index patients

Two patients diagnosed with profound to severe bilateral mixed HL at the ENT department of the State hospital in Salzburg (Austria) were submitted to genetic analysis in order to determine the cause of the observed hearing defect.

**FIGURE 1:**
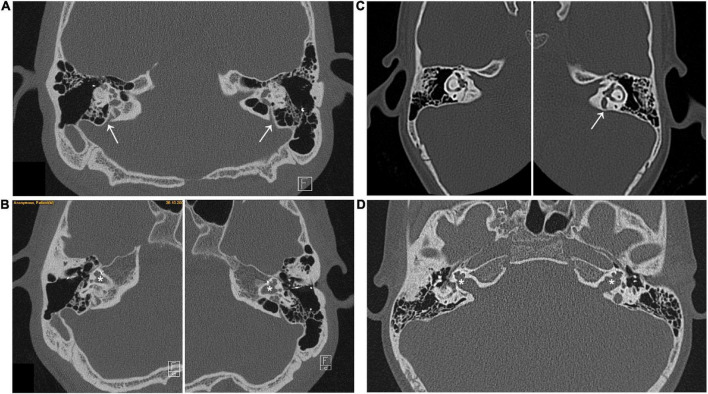
Axial CT scan of the index patients showing both sides. **(A,B)** Patient #569, arrows in panel **(A)** indicate the bilateral enlargement of the vestibular aqueduct (EVA), * in panel **(B)** indicate the bilateral incomplete partition of the cochleae with missing modiolus (IP3), a typical hallmark of POU3F4-related deafness. **(C,D)** Patient #667, arrow in panel **(C)** indicates the EVA on the left side, while on the right side the vestibular aqueduct appears normal; * in panel **(D)** indicate the bilateral IP3.

The first patient (patient ID #569) is a 33-year-old Caucasian male diagnosed with a bilateral severe mixed HL characterized by a progressive loss of hearing from the age of 6. The CT scan of the temporal bone revealed a bilateral EVA and a bilateral IP3 (Figures 1A,B). During cochlear implant surgery, a CSF gusher was observed. Both parents have normal hearing.

The second patient (patient ID #667) is a 19-year-old Caucasian male with profound mixed HL in the right ear and severe mixed HL in the left ear, associated with dizziness. From the CT scan, an IP3 was observed on both sides, while the EVA was present on the left side only (Figures 1C,D) that, interestingly, is the side diagnosed with the less severe degree of HL. A CSF gusher was observed during surgery also with this patient. In addition to the HL, patient #667 was diagnosed externally with cognitive deficits. Both parents and the brother have normal hearing. Clinical findings concerning the two patients presented here are summarized in Table 1. The clinical features displayed by the two patients corresponded to the typical hallmarks associated with genetic defects in *POU3F4*, that are an IP3, EVA, intraoperative CSF gusher, HL often associated with mental deficits and/or vestibular dysfunction (Wang et al., 2021).

Index patient #569 received cochlear implants on both sides, patient #667 on one side, with conventional hearing aid on the other side. All electrodes could be correctly positioned within the cochlea, however, patient #569 needed revision 17 years after primary surgery due to facial nerve stimulation through the implant. Hearing rehabilitation with the cochlear implant in both patients resulted in pure-tone audiometry curve threshold at around 20 dB HL on the implanted side, with 100% numbers and 15% monosyllabics recognition at 65 dB SPL for Freiburger speech audiometry in silence.

### Identification of novel *POU3F4* variants

**FIGURE 2:**
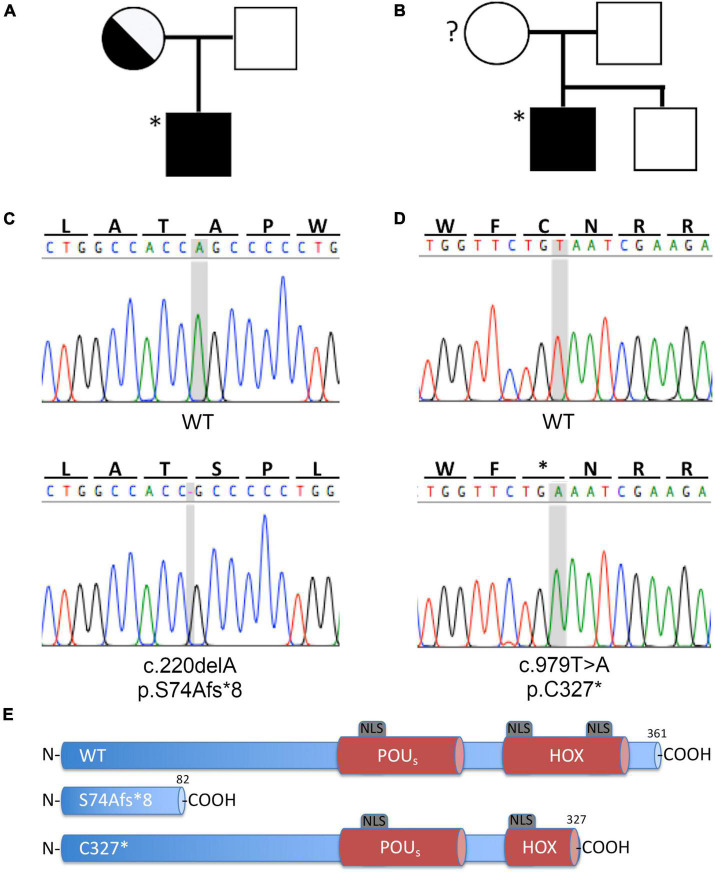
Novel *POU3F4* variants identified in two patients of the Austrian cohort with HL and EVA. **(A,B)** Family pedigree of patients #569 and #667, respectively. The index patients are indicated with an asterisk (*). The genotype of the mother of patient #667 is unknown. **(C,D)** Electropherograms from Sanger sequencing of the gDNA of patients #569 and #667, respectively; the position involved in the sequence variation is shaded in gray. **(E)** Graphic representation of the protein products, compared to the wild type protein; both nucleotide sequence alterations lead to a premature protein truncation. HOX, Homeobox DNA binding domain; NLS, nuclear localization sequence; POU_S_, POU specific DNA binding domain. Protein structure based on information from Lee et al. (2009).

The finding of an IP3 in the two index patients prompted us to analyze *POU3F4*. Due to the presence of an EVA in both patients, all known EVA-related genes were included in the analysis. Genomic DNA was extracted from peripheral blood of index patients and regions of interest were amplified for Sanger sequencing. Two novel sequence variants in the *POU3F4* gene were identified in these patients (Table 2). The variant identified in patient #569 is a deletion of an alanine at position g.5284, c.220, resulting in a frameshift and a premature truncation of the protein at p.82 (g.5284delA, c.220delA, p.S74Afs*8). The variant identified in patient #667 is a conversion of a thymine to an adenine at position g.6045, c.979, resulting in a premature stop codon at position p.327 (g.6045T > A, c.979T > A, p.C327*). Familial pedigrees are shown in Figures 2A,B. The mother of patient #569 is a heterozygote carrier of the mutation identified in the index patient. The genetic status of the mother of patient #667 is unknown. The two sequence variants identified in *POU3F4* are novel, and represent the first report of *POU3F4* mutations in the Austrian population. Electropherograms from the Sanger sequencing and a graphical representation of the protein products resulting from the mutated genes are shown in Figures 2C–E, respectively. Supplementary Table 3 shows the additional genetic findings of index patients #569 and #667. No pathogenic sequence variants were found in the known EVA-related genes *SLC26A4*, *FOXI1*, *KCNJ10* and *GJB2/GJB6*.

**TABLE 2:** Summary of *POU3F4* variants identified in the two index patients.

Patient ID	Sex	*POU3F4* (gDNA)		*POU3F4* (cDNA)		POU3F4 (protein)	
#569	M	g.5284delA	–	c.220delA	–	p.S74Afs*8	–
#667	M	g.6045T > A	–	c.979T > A	–	p.C327*	–

Results from the Sanger sequencing were compared to the following NCBI reference sequences: genomic DNA (RefSeq NG_009936.2), mRNA (RefSeq NM_000307.1) and protein product (RefSeq NP_00298.3). *Denotes the STOP codon.

### Subcellular localization of wild type and mutant POU3F4

**FIGURE 3:**
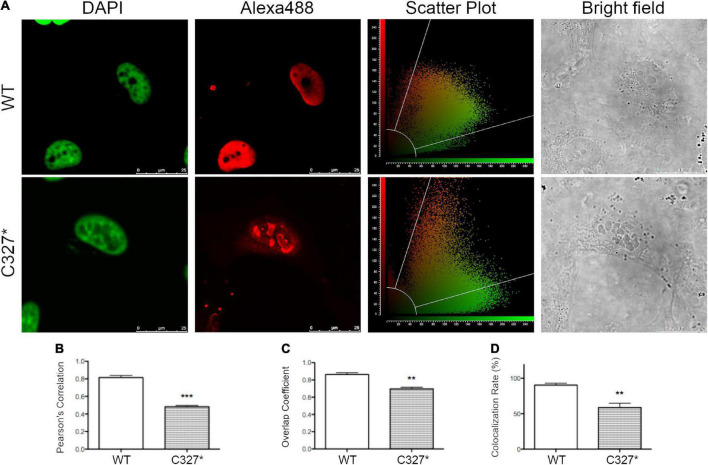
The novel POU3F4 p.C327* protein variant identified in the Austrian cohort shows an altered nuclear localization. **(A)** Confocal imaging of the subcellular localization of wild type POU3F4 and POU3F4 variant p.C327*. Co-localization of the nuclear marker DAPI and POU3F4 with a N-terminal FLAG tag imaged 42 h after transfection in HeLa cells is shown. The corresponding scatter plots graphically display the presence or absence of colocalization of the two signals. **(B–D)** Quantification of the co-localization by Pearson’s correlation coefficient, overlap coefficient and colocalization rate, respectively. ***p < 0.001, **p < 0.01 compared to wild type, unpaired, two-tailed Student’s *t*-test.

The two POU3F4 protein variants identified in the two index patients were reproduced in cell-based assays as fusion proteins with a N-terminal FLAG tag and their expression and subcellular localization was investigated (Supplementary Figure 1 and Figure 3). Imaging was performed in transiently transfected HeLa cells processed for immunocytochemistry with an anti-FLAG antibody. While wild type POU3F4 and POU3F4 variant p.C327* were found to be expressed in the cell nucleus, variant p.S74Afs*8 could not be detected (Supplementary Figure 1), most likely as a result of prompt degradation of this small protein fragment. Although variant p.C327* was exclusively localized in the nucleus, it accumulated in condensed bright spots. Accordingly, co-localization with DAPI gave a Pearson’s correlation coefficient of 0.82 ± 0.02, *n* = 3, for the wild type protein and 0.48 ± 0.02, *n* = 4, for p.C327*, thus denoting the dissimilar distribution pattern of the two proteins (Figure 3).

The exclusive nuclear localization of wild type POU3F4 was confirmed following expression of the fusion protein EYFP-POU3F4 in HeLa cells (Supplementary Figure 2). In contrast, protein variant p.S74Afs*8 showed a localization pattern distributed in the whole cell body, probably as a result of a stabilization of the protein fragment by fusion to EYFP. Concerning POU3F4 variant p.C327*, the localization in condensed bright nuclear spots was confirmed. As the protein truncation takes place within the DNA binding homeodomain near the C-terminus (Figure 2E), this variant retains two out of three predicted nuclear localization sequences (NLS) (Lee et al., 2009), which is probably sufficient to direct the protein to the nucleus. The altered expression pattern of the two variants is consistent with a possible pathogenic role.

### Analysis of the transcriptional activity of wild type and mutant POU3F4 on a synthetic construct

HEK293Phoenix cells were co-transfected with a plasmid vector carrying the CDS of wild type or mutant *POU3F4*, a plasmid vector carrying the CDS of the firefly luciferase driven by a portion of *POU3F4* promoter (-472 bp to + 25 bp, where + 1 denotes the A of the starting codon ATG), and a vector carrying the CDS of the renilla luciferase driven by a CMV promoter, as a normalizer. The portion of *POU3F4* promoter employed here was shown to efficiently drive the transcription of reference genes by POU3F4 in Malik et al. (1996) and more recently confirmed in Lee et al. (2009).

**FIGURE 4:**
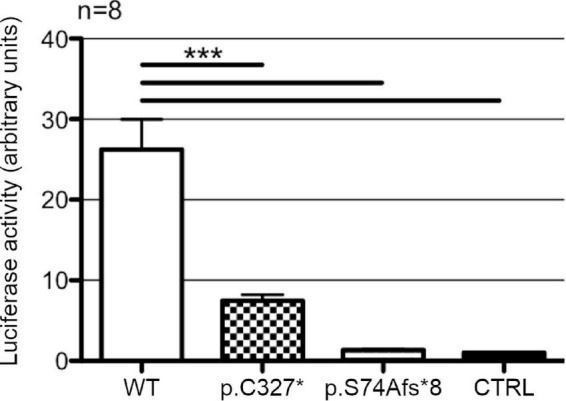
Transcriptional activity of wild type POU3F4 and its protein variants. Luciferase activities in HEK293Phoenix cells co-transfected with the reporter construct containing a section of *POU3F4* promoter region (from –472 to +25 bp) and constructs coding for POU3F4 in its wild type form or reproducing the variants identified in the patients. Wild type POU3F4 could efficiently drive the transcription of the reporter gene, while the mutant POU3F4 proteins could not. *n* represents the number of independent transfections. ***p < 0.001 compared to wild type, one-way ANOVA with Bonferroni’s post-test.

Wild type POU3F4 could efficiently drive the transcription of the reporter gene, showing a 26-fold increase in the firefly luciferase signal in comparison to the control (Figure 4). POU3F4 p.C327* led to a slight increase in the transcription of the reporter gene over the basal level that was, however, not statistically significant (+7.5-fold, *p* > 0.05). POU3F4 p.S74fs*8 showed no significant increase in the transcription of the reporter gene and was indistinguishable from the control.

### Transcriptome analysis in cells overexpressing wild type or mutant POU3F4

**FIGURE 5:**
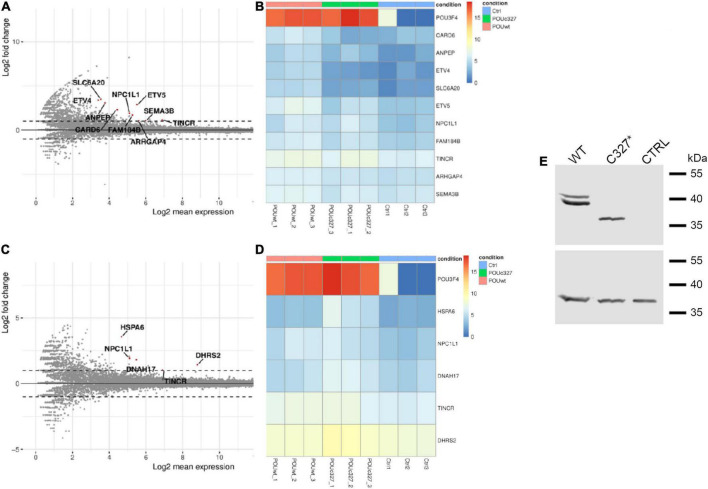
Transcriptome analysis (RNAseq) of total RNA extracted from POU3F4-overexpressing HEK293Phoenix cells. The RNAseq analysis was performed on RNA extracted from three independent subcultures of HEK293Phoenix cells transiently transfected with wild type POU3F4, POU3F4 p.C327*, or a control vector encoding solely for the transfection marker EGFP. Volcano plots showing differentially regulated transcripts in wild type POU3F4-transfected cells **(A)** and POU3F4 p.C327*-transfected cells **(C)** compared to control cells are shown. Each dot in the plot represents a transcript identified in the analysis. Above the line indicated with a “0” are the transcripts that were upregulated in transfected cells versus the control cells, below the 0 are the downregulated transcripts. Red dots represent transcripts showing a statistically significant upregulation. Heatmap of the top differentially expressed transcripts (DESeq2 results with independent filtering, adj. *p*-value <0.05, logFC ≥ ± 1) in wild type POU3F4-transfected cells **(B)** and POU3F4 p.C327*-transfected cells **(D)** compared to control. **(E)** Top panel: western blot on total protein extracts from cells overexpressing wild type POU3F4 or POU3F4 p.C327*; control cells were transfected with an empty vector. Bottom panel, the signal of the housekeeping protein GAPDH served as the loading control.

HEK293Phoenix cells were transiently transfected with plasmid vectors carrying the CDS of wild type POU3F4, POU3F4 variant p.C327*, or an empty vector (negative control). Total RNA extracted from the transfected cells was submitted to RNA sequencing (RNAseq). The expression profile of cells transfected with wild type POU3F4 was compared to the negative control (Figures 5A,B) and to cells transfected with POU3F4 p.C327* (Figures 5C,D). As expected, *POU3F4* transcript was found significantly upregulated in wild type and mutant POU3F4-transfected cells compared to control cells, as a result of a successful transfection with similar efficiency for both plasmid vectors (Figures 5B,D). Expression of wild type POU3F4 and POU3F4 p.C327* was also confirmed by Western blot (Figure 5E). Among the transcripts differentially expressed in wild type POU3F4-transfected cells compared to control, a highly significant upregulation (log2-fold change = 3.488, false discovery rate-adjusted *p*-value = 0.001281) was detected for the amino acid transporter solute carrier family 6 (proline imino transporter), member 20 (*SLC6A20*, SIT1, XTRP3). Further transcripts that were significantly upregulated also include those of the caspase recruitment domain-containing protein *CARD6* (log2-fold change = 2.31, adj. *p*-value = 0.002), the aminopeptidase *ANPEP* (log2-fold change = 3.07, adj. *p*-value = 0.0046), the transcription activator *ETV4* (log2-fold change = 3.37, adj. *p*-value = 0.0029), and the DNA binding protein *ETV5* (log2-fold change = 2.88, adj. *p*-value = 3.87*10-9), among others (Figures 5A,B and Supplementary Table 4). Importantly, these transcripts were not upregulated in cells overexpressing the truncated form of POU3F4 (Figures 5C,D and Supplementary Table 4). *SLC6A20* was chosen for further investigations because of the highest fold-change upregulation resulting from the RNAseq analysis.

**FIGURE 6:**
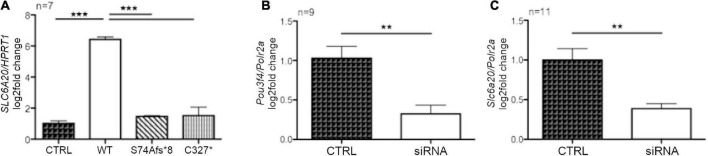
POU3F4 regulates the transcription of *SLC6A20*. **(A)** HEK293Phoenix cells were transiently transfected with plasmid vectors coding for wild type POU3F4 or the indicated POU3F4 variants. Cells transfected with an empty vector served as negative control. Total RNA was reverse-transcribed and subjected to RT-qPCR. The levels of the *SLC6A20* transcript were normalized to those of the housekeeping transcript *HPRT1*. The *SLC6A20* transcript was upregulated in HEK293Phoenix cells overexpressing wild type POU3F4, but not in cells overexpressing the POU3F4 variants. ***p < 0.001, one-way ANOVA with Bonferroni’s post-test. **(B,C)** RT-qPCR on mRNA extracted from MEFs transfected with a combination of siRNAs designed to silence *Pou3f4* or with a scrambled control siRNA (control, CTRL). Expression levels of *Pou3f4* and *Slc6a20* were normalized to those of the housekeeping transcript *Polr2a.* Silencing of *Pou3f4* led to reduced *Slc6a20* expression. n represents the number of biological replicates. **p < 0.01, two-tailed, unpaired Student’s *t*-test.

The results of the RNAseq were validated by RT-qPCR in HEK293Phoenix cells transiently overexpressing the POU3F4 variants (wild type, pS74Afs*8, or p.C327*), compared to a negative control (cells transfected with an empty vector). The transcript of *SLC6A20* was upregulated in cells overexpressing wild type POU3F4 (log2-fold change = 6.43 ± 0.15, *p* < 0.001, Figure 6A). The upregulation of *SLC6A20* was not observed in cells overexpressing any of the two protein variants (no statistically significant differences compared to the negative control were observed, Figure 6A). In order to further confirm the functional interaction between Pou3f4 and the candidate target, the expression of *Slc6a20* has been evaluated in cells transfected with a combination of siRNAs designed to silence *Pou3f4* (Supplementary Table 5). MEFs have been chosen as they contain detectable basal levels of both transcripts and represent a cell type similar to the fibrocytes of the spiral ligament of the cochlea where POU3F4 is physiologically expressed. The transfection with the selected combination of siRNAs against *Pou3f4* led to a significant reduction in the expression of the target transcript, if compared to control cells transfected with a scrambled siRNA (Figure 6B, *Pou3f4* silencing: 69%, *p* < 0.01). In correspondence with the decreased expression of *Pou3f4*, a significant decrease in the expression of the transcript of the putative target gene *Slc6a20* was observed when compared to the negative control (Figure 6C, *Slc6a20* expression reduction: 70%, *p* < 0.01).

### SLC6A20 is expressed in the mouse cochlea

**FIGURE 7:**
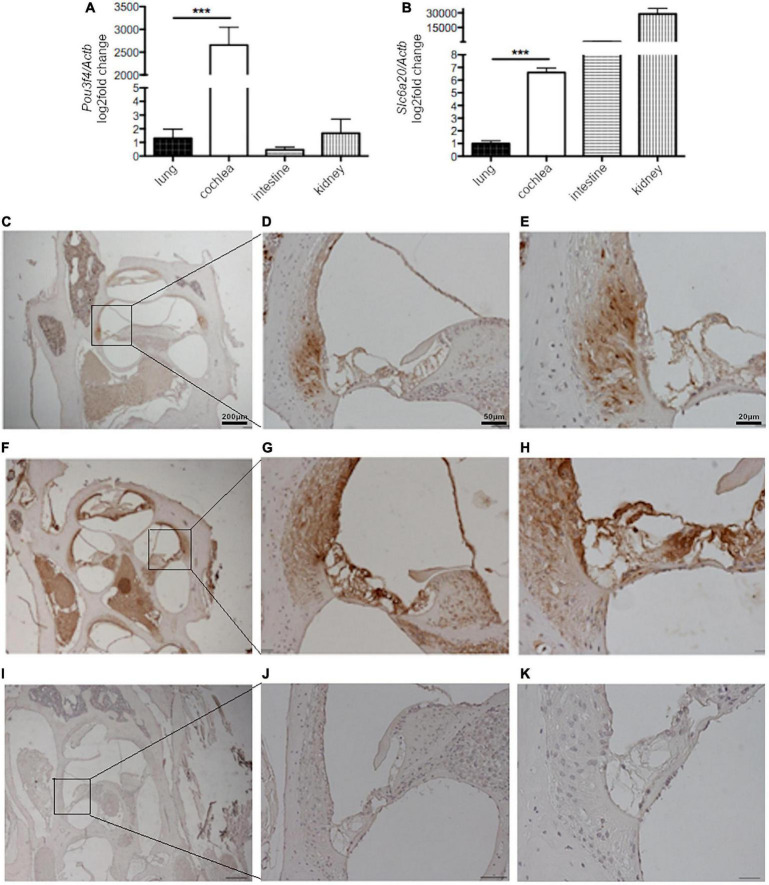
*Slc6a20* and *Pou3f4* transcripts and proteins are co-expressed in the mouse cochlea. RT-qPCR on mRNA extracted from whole mouse cochlea, lung, intestine, and kidney and expression of **(A)** *Pou3f4* and **(B)** *Slc6a20* transcripts relative to the housekeeping transcript *Actb*. *n* = 5 represents the number of biological replicates. ***p < 0.001, two-tailed, unpaired Student’s *t*-test. Immunostaining of tissue slices from mouse cochlea with **(C–E)** an anti-SLC6A20 primary antibody, **(F–H)** an anti-POU3F4 primary antibody, and **(I–K)** only the secondary antibody as the negative control. SLC6A20 was clearly localized in the spiral ligament. POU3F4 was more widely distributed in different structures of the cochlea, including the spiral ligament.

So far, no evidence was available regarding the expression of SLC6A20 in the inner ear. We determined the expression of *Pou3f4* and *Slc6a20* in the whole cochlea of C57BL/6J mice by RT-qPCR. *Pou3f4* transcript was highly expressed in the cochlea, while it was detectable at very low levels in other mouse tissues such as the lung, liver and small intestine (Figure 7A), in agreement with previous findings (Minowa et al., 1999; Uetsuka et al., 2015). *Slc6a20* transcript was highly expressed in the kidney and small intestine as already reported (Takanaga et al., 2005), almost undetectable in lung but, most importantly, clearly detectable in mouse cochlea (Figure 7B). These results show that both *Pou3f4* and *Slc6a20* transcripts are expressed in the mouse cochlea.

To confirm the co-expression of POU3F4 and SLC6A20 proteins in the cochlea, immunostaining on slices from the cochlea of wild type mice was performed. Immunohistochemistry with an anti-SLC6A20 antibody showed a protein expression that appeared to be specific when compared to sections stained with the secondary antibody only. SLC6A20 was found in distinct cochlear structures, with prominent expression in the spiral ligament (Figures 7C–E). A staining with anti-POU3F4 antibodies confirmed the expression of this transcription factor in the same area of the spiral ligament, as well as in other structures of the cochlea including the *stria vascularis*, as reported in the literature (Minowa et al., 1999; Uetsuka et al., 2015; Figures 7F–H). As mentioned above, a negative control staining with the secondary antibody only was performed (Figures 7I–K), and gave no signal. These data strongly suggest that POU3F4 and SLC6A20 are co-expressed in the spiral ligament.

## Discussion

Typical hallmark of POU3F4-related HL is the presence of an incomplete partition of the cochlea, defined as IP3, which is observed in combination with an EVA in about 50% of cases (Roesch et al., 2021). Two patients from our EVA cohort displayed these typical features and were submitted to DNA analysis for the determination of the genetic defect responsible for the observed phenotype. A defect in the coding sequence of *POU3F4* was identified by Sanger sequencing in both patients, confirming the correlation between IP3 and EVA with *POU3F4* pathogenic sequence alterations.

When present, EVA in *POU3F4* patients often differs from that classically described in DFNB4/Pendred syndrome. Rather than conical, vestibular aqueducts in *POU3F4* patients are large and symmetrical and become cystic or enlarged from the middle parts to the ends near the vestibule but not at the operculum (Roesch et al., 2021). We observed this EVA variant in index patient #667 (Figure 1C), while EVA in patient #569 appeared rather similar to that described in the context of DFNB4/Pendred syndrome (Figure 1A).

Both gene variants identified in this study (c.220delA and c.979T > A, Figure 2) were novel, and therefore no related functional information was available. In order to determine or exclude the pathogenicity of these gene variants, analysis of the subcellular localization and transcriptional activity of the corresponding protein products was performed.

Both gene variants are predicted to result in a premature stop codon and a truncation of the protein product. The short variant (p.S74Afs*8) lacks both DNA binding domains and the three predicted NLSs; the longer variant (p.C327*) results from the generation of a stop codon in the middle portion of the second DNA binding domain and therefore the resulting protein product still includes the first DNA binding domain and two of the three predicted NLSs (Figure 2E). We observed a defective expression and subcellular localization of both variants when compared to the wild type protein. As expected for a transcription factor, the wild type POU3F4 protein localized exclusively in the nuclear compartment. In contrast, the two variants showed a disturbed subcellular localization, with the longer variant (p.C327*) localizing in the nucleus but, other than the wild type protein, accumulating in bright spots possibly corresponding to the nucleoli (Figure 3). The two NLSs that are still present in this protein variant were obviously sufficient for the nuclear targeting. However, the premature truncation of the polypeptide could have unmasked hydrophobic regions, thus causing incorrect folding of the aberrant protein product and leading to its aggregation and condensation in the nucleus. As expected, the shorter variant (p.S74Afs*8), missing a large portion of the polypeptide, could not be detected, probably as a result of prompt degradation (Supplementary Figure 1).

An aberrant cellular distribution of two POU3F4 variants similarly leading to truncated protein products was formerly described. These variants were identified in a Jewish family originating from Bulgaria (p.Ile285Argfs*43) and in a N-ethyl-N-nitrosourea (ENU)-induced mutant mouse (p.Cys300*). Both protein variants were largely mislocalized to the cytoplasm in cell-based assays, with a smaller portion remaining in the nucleus and an overall substantial reduction of protein abundance (Parzefall et al., 2013). As protein variant p.C327* was expressed exclusively in the nucleus in our assays (Figure 3), it is likely that amino acids 300-327 are essential for nuclear targeting, while downstream C-terminal regions might be important for proper protein folding.

The defective expression and subcellular localization of the protein variants identified in our patients was reflected in their altered transcriptional activity. Both protein variants showed a severe impairment in the transcription efficiency when compared to the wild type, with the longer variant displaying a slightly higher activity than the short variant, though not statistically significant (Figure 4). The longer variant, although localized within the nucleus, failed to efficiently drive the transcription of the reporter gene. These results indicate that the C-terminal portion of the HOX domain is essential for the transcriptional activity of POU3F4. These functional and molecular aspects, together with the typical inner ear malformations and the absence of pathogenic sequence alterations in other known EVA-related genes (Supplementary Table 3) strongly support the pathogenicity of protein variants p.S74Afs*8 and p.C327*, unequivocally identifying *POU3F4* defects as the genetic determinant for the hearing deficit observed in the two index patients.

Genotype-phenotype correlation in these two patients based on our functional data is not straightforward. Even assuming that variant p.C327* might retain some residual activity compared to p.S74Afs*8, it has to be noted that this variant was identified in the patient with the more severe clinical phenotype in terms of severe to profound hearing loss, which was associated with vestibular dysfunction and cognitive impairment. However, it also has to be noted that this patient presented with unilateral EVA, while the patient harboring the p.S74Afs*8 variant had bilateral EVA. Whether residual or loss of transcriptional activity of POU3F4 variants can be linked to unilateral or bilateral EVA, respectively, requires further investigation.

The molecular mechanism of POU3F4-related HL is largely unknown and its transcriptional targets are only partially characterized. Only few studies could reveal functional targets of POU3F4 in the hearing organ. Coate and colleagues showed that the expression of the receptor tyrosine kinase EphA4 is decreased in *Pou3f4* knockout mice and that POU3F4 binds *EphA4* regulatory elements (Coate et al., 2012), which is important for spiral ganglion axon fasciculation. The same group showed that POU3F4 and the Eph receptor transmembrane ligand Ephrin-B2 exhibit a common spatio-temporal expression pattern during organogenesis (Raft et al., 2014). Although these studies provided evidence for a role of POU3F4 in the development of the spiral ganglion and bony structures of the middle ear, the link with the cochlear malformations typical of POU3F4-related deafness remained unexplained, pointing to the possible existence of additional cochlear targets. Our RNAseq analysis delivered several additional novel putative transcriptional targets of POU3F4 (Figure 5 and Supplementary Table 4) as well as targets that are differentially upregulated by wild type and p.C327* POU3F4 (Supplementary Table 4). *CARD6*, *ANPEP*, *ETV4*, *SLC6A20*, *ETV5*, F*AM184B*, *ARHGAP4*, and *SEMA3B* are found significantly upregulated by wild type POU3F4 compared to control (Figures 5A,B) and not by POU3F4 p.C327* compared to control (Figures 5C,D), leading to speculate that loss or reduction of expression of one or more of these targets might be pathogenic. Of these, *CARD6*, *ETV4*, *SLC6A20*, and *ETV5* are found differentially expressed when comparing POU3F4 p.C327* to the wild type (Supplementary Table 4), and might represent the strongest candidates to be linked to the disease phenotype.

Analysis of the targets upregulated by POU3F4 p.C327* compared to control cells (Figures 5C,D and Supplementary Table 4) revealed an overall loss of function of POU3F4 p.C327* compared to the wild type, including loss of transcriptional activity on all above mentioned targets. However, a limited number of targets still resulted upregulated by POU3F4 p.C327*. Of these targets, some (*NPC1L1* and *TINCR*) were also upregulated by the wild type to a similar, limited extent - the log2 fold change is <2 in both cases. Therefore, the significance of these findings is currently unclear. Genes that were found significantly upregulated by POU3F4 p.C327* and not by the wild type are *HSPA6*, *DNAH17*, and *DHRS2*. Heat Shock Protein Family A Member 6 (*HSPA6*; OMIM *140555) is a stress-induced heat-shock gene encoding a basic 70-kD protein. Dynein, Axonemal, Heavy Chain 17 (*DNAH17*; OMIM *610063) is a microtubule-associated motor protein expressed in adult testis of which mutations cause spermatogenic failure. *DHRS2* (OMIM *615194) encodes a member of the short-chain dehydrogenases/reductases that reduces proliferation, migration and invasion of cancer cells and well as the production of reactive oxygen species in cancer. With the exception of *DNAH17*, of which the significance is currently unclear in this context, the other two genes might have been upregulated following cellular stress and increased oxidative stress as a consequence of the overexpression of a misfolded protein.

Among the targets significantly upregulated following wild type POU3F4 overexpression, the amino acid transporter SLC6A20 was selected for further investigations. The upregulation of this specific target could be confirmed by RT-qPCR (Figure 6A). Interestingly, neither of the two POU3F4 variants identified resulted in upregulation of the *SLC6A20* transcript, further supporting their pathogenicity. Accordingly, silencing of the endogenous *Pou3f4* transcript in a cell model reminiscent of the native cells expressing POU3F4 within the inner ear led to reduced expression of *Slc6a20* (Figures 6B,C).

Function and expression of SLC6A20 (OMIM *605616), formerly called Sodium/Imino-acid Transporter 1 (SIT1), have been first characterized by Takanaga et al., who found that this transporter leads to proline uptake in a Na^+^– and voltage-dependent, Cl^–^-stimulated, pH-independent manner and is expressed on the apical membrane of epithelial cells in various portions of the intestine as well as the kidney proximal tubule (Takanaga et al., 2005). According to this seminal study, *SLC6A20* gene variants have been later found to contribute to forms of autosomal dominant hyperglycinuria (phenotype MIM number 138500) as well as digenic iminoglycinuria (phenotype MIM number 242600) (Broer et al., 2008). Iminoglycinuria is a rare autosomal recessive disorder characterized by increased urinary excretion of proline, hydroxyproline, and glycine that has been described in association with mental retardation and sensorineural hearing loss in some cases (Goyer et al., 1968; Swarna et al., 2004). Furthermore, SLC6A20 has been shown to be involved in the transport of other amino acids beside proline, namely hydroxyproline, N-methylaminoisobutyric acid, and betaine (Kowalczuk et al., 2005; Takanaga et al., 2005). It is therefore conceivable that a defective imino acid transport resulting from reduction of expression SLC6A20 in the inner ear as a consequence of POU3F4 loss of function might lead to sensorineural hearing loss, similarly to what was observed in the context of *SLC6A20* pathogenic gene variations.

In addition to pathogenic gene variations, also a reduction of expression of *SLC6A20* in the kidney might in principle lead to iminoglycinuria. However, this defect might be compensated by redundancy of imino acid transporters in the kidney (Verrey et al., 2009).

No evidence of SLC6A20 expression in the inner ear was available so far. In the present study we could show the presence of *Slc6a20* transcript in the mouse cochlea and confirm the expression of *Pou3f4* (Figures 7A,B), as already reported in the literature (Minowa et al., 1999). Moreover, we showed here for the first time a spatial co-expression of SLC6A20 and POU3F4 proteins in the same regions of the mouse cochlea and specifically in the area of the spiral ligament (Figures 7C–H). Spatial co-expression is a necessary prerequisite for any functional interaction between the two genes/gene products and therefore represents a crucial finding guiding further investigations in the pathophysiological role of POU3F4 and SLC6A20 in the inner ear.

The role of solute transporters and channels is crucial for the function of the auditory system. Ion channels and transporters ensure the maintenance of the endolymph ion composition and pH, which are essential for the transmission of the auditory signal and preservation of the inner ear structures (Lang et al., 2007). Potassium channels permit the recycling of potassium ions from the endolymph and through the organ of Corti, transducing the mechanical sound signal into an electric signal and leading to the release of neurotransmitter and action potentials generation in the efferent auditory nerve terminations (Mittal et al., 2017). Pendrin-mediated Cl^–^/HCO_3_^–^ exchange contributes to the osmotic balance and pH regulation of the endolymphatic fluid (Choi et al., 2011). Transporters for organic solutes are also active in the cochlea, contribute to the homeostasis of this organ and allows for the exchange of compounds between the cochlea and the vasculature (Uetsuka et al., 2015). Here we show that the proline imino transporter SLC6A20 seems to be exclusively expressed in a discrete region of the mouse cochlea within the spiral ligament, corresponding to the type II fibrocytes (Figures 7D,E). Cochlear fibrocytes form a connective tissue syncytium within the spiral ligament that, together with the *stria vascularis*, plays a fundamental role in the cochlear potassium recycling and maintenance of the endocochlear potential. Five distinct types of fibrocytes can be identified based on their anatomical position within the spiral ligament and their structural characteristics, including the density of cytoplasm, content of organelles, and the extension of the plasma membrane folding. Type II fibrocytes are connected to type I fibrocytes via the connexin network and express a battery of ion channels and transporters, including particularly elevated levels of the Na^+^/K^+^ ATPase, the inwardly rectifying K^+^ channel Kir5.1, the Na^+^-K^+^-2Cl^–^ cotransporter-1 (NKCC1) and the glutamate-aspartate transporter SLC1A3/GLAST (Furness et al., 2009; Furness, 2019). These and our findings identify fibrocytes, and specifically type II fibrocytes, as fundamental players of the amino acids homeostasis within the cochlea, beside K^+^ recycling.

Another amino acid transporter, SLC7A8, was previously detected in key structures of the inner ear including the spiral ligament, mostly in type I fibrocytes (Espino Guarch et al., 2018). Espino Guarch and colleagues have shown a correlation between hypofunctional variants of SLC7A8 and age-related HL associated to morphological defects in the spiral ligament and *stria vascularis* in a mouse model (Espino Guarch et al., 2018). The defects observed included a reduction in the number of fibrocytes in the spiral ligament and loss of gap junction expression. Interestingly, these alterations are reminiscent of those observed in *Pou3f4* knockout mice (Kidokoro et al., 2014). These findings and the findings presented here point to the relevance of amino acid homeostasis for the auditory function. Further investigations are necessary in order to define how a possible lack or reduction of SLC6A20 expression could lead to inner ear malformations and HL in POU3F4-related deafness.

## Data availability statement

The datasets presented in this study can be found in online repositories. The names of the repository/repositories and accession number(s) can be found below: www.ncbi.nlm.nih.gov, GSE211647.

## Ethics statement

The studies involving human participants were reviewed and approved by the Ethics Committee of the County of Salzburg, Austria. The patients/participants provided their written informed consent to participate in this study. Ethical review and approval was not required for the animal study because no animal treatment was performed. Animal sacrifice for tissue harvesting with no animal treatment does not require approval of an animal protocol according to local regulations.

## Author contributions

EB, AS, and SD conceived the study. EB, SR, LA, and SD designed the experiments. SR and GR contributed the clinical images and data. EB and ES performed the experiments and analyzed the data. EB wrote the original draft. SD edited the manuscript. AS and SD contributed equally to this work and share senior authorship. All authors participated in the interpretation of results, reviewed and approved the final version of the manuscript.

## Footnotes

1 Available online: https://www.wma.net/policies-post/wma-declaration-of-helsinki-ethical-principles-for-medical-research-involving-human-subjects/.
