# Supplementary material for: Novel POU3F4 variants identified in patients with inner ear malformations exhibit aberrant cellular distribution and lack of SLC6A20 transcriptional upregulation
Source: Front Mol Neurosci. 2022 Sep 29;15:999833. doi: 10.3389/fnmol.2022.999833 (PMC9558712; doi:10.3389/fnmol.2022.999833)
Supplement: Supplementary file 1 [file Data_Sheet_1.docx]

**Table S1. Primers for end-point PCR and Sanger sequencing.**

| **Gene** | **PCR primer sequence** | **Amplicon size**  **(bp)** | **Sequencing primer sequence** | **Target region** |
| --- | --- | --- | --- | --- |
| ***POU3F4*** | fwd, 5´ TATTGGCCGGGCTTACTCC 3´ | 1662 | fwd, 5´ TATTGGCCGGGCTTACTCC 3´ | Exon 1 |
|  | rev, 5´ AGATCCCAGCTTGGACTGC 3´ |  | rev, 5´ GTCTCCTCGTCGGAGTGATC 3´ |  |
|  |  |  | fwd, 5´ AACGTGTACTCGCAGCCTG 3´ | Exon 2 |
|  |  |  | rev, 5´ AGATCCCAGCTTGGACTGC 3´ |  |
| ***GJB2*** | fwd, 5´ CACGTTCAAGAGGGTTTG 3´ | 987 | Same as for PCR | Exon 1 |
|  | rev, 5´ TGAGCCTTGACAGCTGAGC 3´ |  |  |  |
| ***FOXI1*** | fwd, 5´ TAGCATGTCATTAGTGGG 3´ | 3054 | fwd, 5´ TAGCATGTCATTAGTGGG 3´ | Exon 1 |
|  | rev, 5´AACAGAAGCCTTAACCAG 3´ |  | rev, 5´ GCTGAAACCCAAATCTTC 3´ |  |
|  |  |  | fwd, 5´ CCTCCATTTCTCAGTCCC 3´ | Exon 2 |
|  |  |  | rev, 5´AACAGAAGCCTTAACCAG 3´ |  |
| ***KCNJ10*** | fwd, 5´ AGTAGCTGGGACTACAGGCGCA 3´ | 1624 | fwd, 5’ TAATTCCTCCCTCCCATG 3´ | Exon 1 |
|  | rev, 5´ AAAGGGGACAGTGGGAGGCGAA 3´ |  | rev, 5´ CAGGGCATTGGAAGAGAG 3´ |  |
|  |  |  | fwd, 5´ GATCCGAGTTGCCAATATGC 3´ |  |
|  |  |  | rev, 5´ CACATTGACCTGGTTGAGCC 3´ |  |

**Table S2. Primers for real-time PCR.**

| **Gene** | **Primer sequence** | **Target region** |
| --- | --- | --- |
| ***POU3F4*** | 5’ GAG ACG CCA ACC TCT GAT G 3’ | Exon 1 |
|  | 5’ CGA GAA CAC GTT ACC ATA CAG T 3’ |  |
| ***SLC6A20*** | 5’ CAG ACA GCA AGA TCA CTT CCAG 3’ | Exon 8-9 |
|  | 5’ CAG CCT CCA TCG TGA ACA 3’ |  |

**Table S3. Genetic findings of index patients #569 and #667 in EVA-related genes.** The minor allele frequency (MAF) is given according to 1000G or, when not available, GnomAD**, retrieved at: <https://www.ncbi.nlm.nih.gov/snp/>. Clinical significance is given according to ClinVar (https://www.ncbi.nlm.nih.gov/clinvar/), accessed on the 01.06.2022. “Not assessed” indicates that the variant is not reported in ClinVar. Reference sequences were NG_008358.1, NM_004004.6 and NP_003995.2 for *GJB2*, NG_012068.2, NM_012188.5 and NP_036320.2 for *FOXI1*, NG_016411.1, NM_002241.5 and NP_002232.2 for *KCNJ10*, NG_008489.1, NM_000441.2 and NP_000432.1 for *SLC26A4*.

|  | ***gDNA*** | ***cDNA*** | **Protein** | **SNP ID** | **MAF** | **Clinical significance** | **Genotype** | **Position** |
| --- | --- | --- | --- | --- | --- | --- | --- | --- |
| ***GJB2*** | | | | | | | |  |
| #569 | *WT* | *WT* | WT |  |  |  |  |  |
| #667 | *WT* | *WT* | WT |  |  |  |  |  |
| ***GJB6* del(GJB6-D13S1830) and del(GJB6-D13S1854)** | | | | | | | |  |
| #569 | not detected | | | | | | |  |
| #667 | not detected | | | | | | |  |
| ***FOXI1*** | | | | | | | |  |
| #569 | *g.5690T>C* |  |  | rs2277945 | C=0.2614 | Not assessed | Heterozygous | Intron 1 |
|  | *g.7606T>C* | *c.1044T>C* | p.Tyr348= | rs10063424 | T=0.9295 | Benign | Homozygous | Exon 2 |
| #667 | *g.7606T>C* | *c.1044T>C* | p.Tyr348= | rs10063424 | T=0.9295 | Benign | Homozygous | Exon 2 |
| ***KCNJ10*** | | | | | | |  |  |
| #569 | *g.33450C>T* | *c.811C>T* | p.Arg271Cys | rs1130183 | A=0.01478 | Benign, likely benign | Heterozygous | Exon 1 |
| #667 | *WT* | *WT* | WT |  |  |  |  |  |
| ***SLC26A4*** | | | | | | | |  |
| #569 | g.7543A>T |  |  | rs2248464 | T=0.7646 | Not assessed | Homozygous | Intron 2 |
|  | g.7549C>T |  |  | rs2248465 | T=0.6585 | Not assessed | Homozygous | Intron 2 |
|  | g.28034G>T |  |  | rs2395911 | T=0.7071 | Not assessed | Heterozygous | Intron 8 |
|  | g.47875T>A |  |  | rs982662 | A=0.9998 | Not assessed | Homozygous | Intron 17 |
|  | g.48138T>C |  |  | rs2188562 | C=0.8287 | Not assessed | Heterozygous | Intron 17 |
|  | g.48487C>T |  |  | rs6955309 | T=0.4776 | Not assessed | Heterozygous | Intron 17 |
|  | g.60680G>A | *c.*868G>A* |  | rs2712218 | A=0.2414 | Benign | Heterozygous | 3´ UTR |
| #667 | *g.5122T>C* | *c.-103T>C* |  | rs60284988 | C=0.002089** | Uncertain significance | Heterozygous | 5´ UTR |
|  | *g.5452T>C* |  |  | rs546225214 | C=0.0004 | Not assessed | Heterozygous | Intron 1 |
|  | *g.7543A>T* |  |  | rs2248464 | T=0.7646 | Not assessed | Homozygous | Intron 2 |
|  | *g.7549C>T* |  |  | rs2248465 | T=0.6585 | Not assessed | Homozygous | Intron 2 |
|  | *g.18828C>T* |  |  | rs1327200615 | T=0.000014** | Not assessed | Heterozygous | Intron 5 |
|  | *g.19045T>G* |  |  | rs3817613 | G=0.6969 | Not assessed | Heterozygous | Intron 5 |
|  | *g.34481C>T* |  |  | rs780920429 | T=0.000008** | Not assessed | Heterozygous | Intron 9 |
|  | *g.48137C>A* |  |  | rs982664 | A=Not assessed | Not assessed | Heterozygous | Intron 17 |
|  | *g.48138T>C* |  |  | rs2188562 | C=0.8287 | Not assessed | Homozygous | Intron 17 |
|  | *g.48487C>T* |  |  | rs6955309 | T=0.4776 | Not assessed | Heterozygous | Intron 17 |
|  | *g.60680G>A* | *c.*868G>A* |  | rs2712218 | A=0.2414 | Benign | Heterozygous | 3´ UTR |
|  | *g.60935A>G* | *c.*1123A>G* |  | rs141341508 | G=0.0008 | Uncertain significance | Heterozygous | 3´ UTR |

**Table S5. Oligonucleotides for the silencing of *Pou3f4* in Mouse Embryonic Fibroblasts.** An equimolar combination of siRNA1, siRNA2 and siRNA3 was employed for the transfection.

| **Name** | **Sequence** |
| --- | --- |
| **siRNA1 POU3F4** | 5’ GGA AAC ACA UAC UCU CUC ATT 3’ |
| **siRNA2 POU3F4** | 5’ ACA CAU ACU CUC UCA UUC ATT 3’ |
| **siRNA3 POU3F4** | 5’ CGA AGA GAG UUA UUG AUG ATT 3’ |
| **Scrambled siRNA (control)** | 5’ AUU CCA UUA ACG AAC GCA CTT 3’ |

**Supplemental Figure Legends**

**Figure S1. The two novel POU3F4 protein variants identified in the Austrian cohort show an altered cellular expression.** Confocal imaging of POU3F4 variants with a N-terminal FLAG tag 42 hours after transfection in HeLa cells. Immunocytochemistry was performed with an anti-FLAG antibody (red) and nuclei have been counterstained with DAPI (green). The image is representative of three independent transfections.

**Figure S2: The two novel POU3F4 protein variants identified in the Austrian cohort show an altered subcellular localization.** Co-localization of the nuclear marker DAPI (green) and fusion proteins EYFP-POU3F4 (red) imaged by confocal microscopy 42 hours after transfection in HeLa cells. The corresponding scatter plots graphically display the presence or absence of colocalization of the two signals. The image is representative of three independent transfections.
