# Supplementary figures and images for: Novel POU3F4 variants identified in patients with inner ear malformations exhibit aberrant cellular distribution and lack of SLC6A20 transcriptional upregulation
Source: Front Mol Neurosci. 2022 Sep 29;15:999833. doi: 10.3389/fnmol.2022.999833 (PMC9558712; doi:10.3389/fnmol.2022.999833)

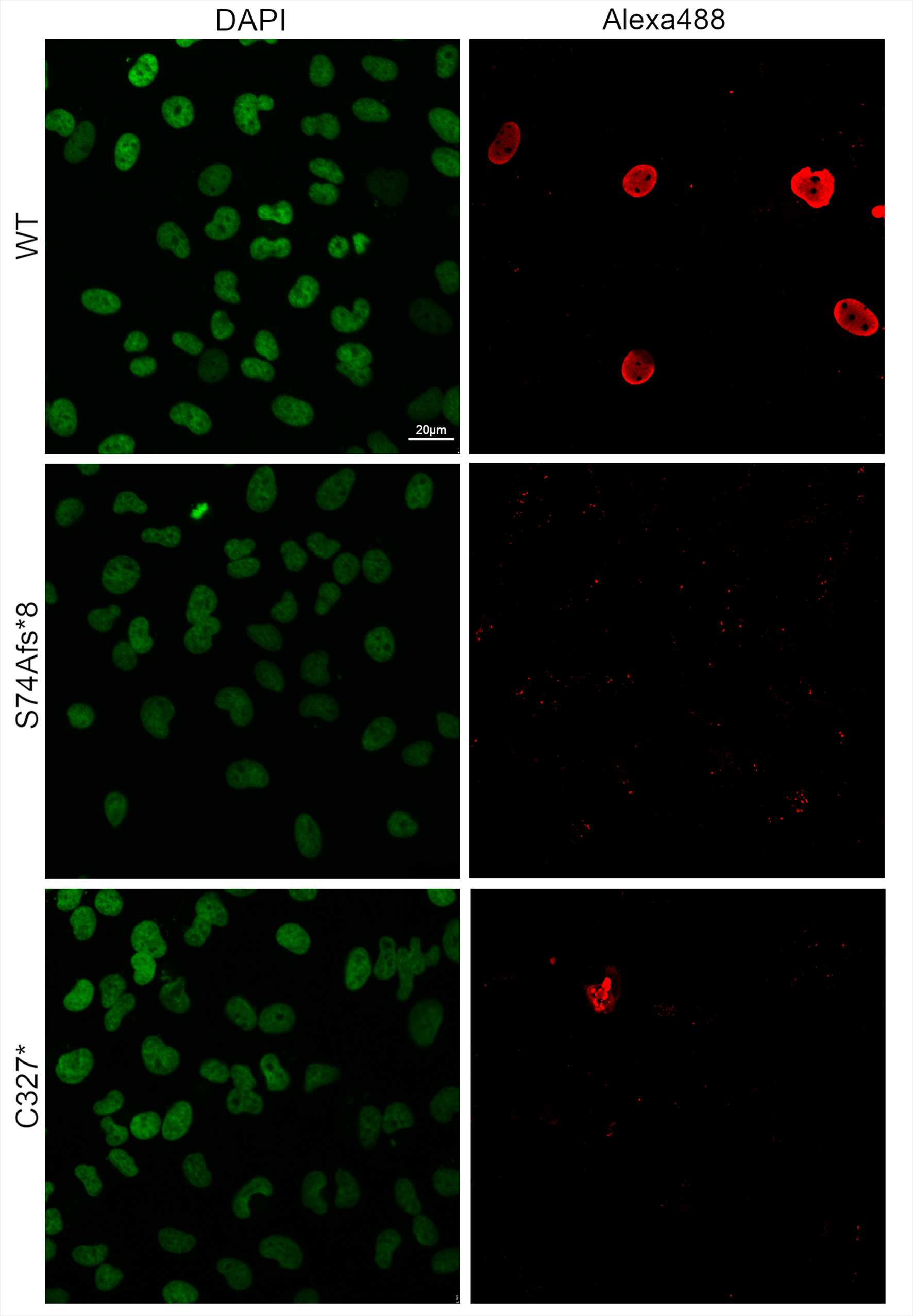

Supplement: Supplementary file 3 [file Image_1.tif]

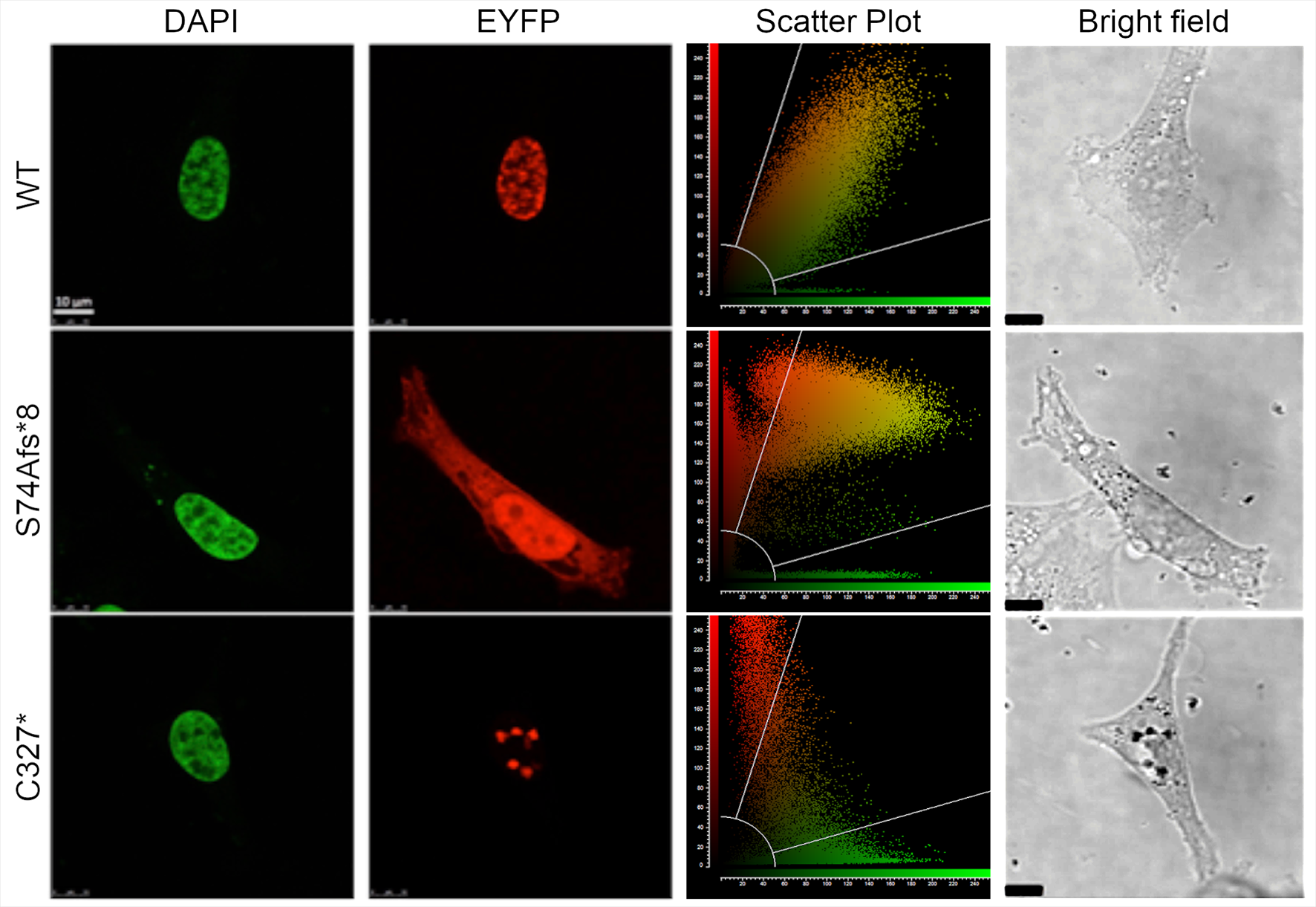

Supplement: Supplementary file 4 [file Image_2.tif]
